# Secondary tension pneumothorax in a COVID-19 pneumonia patient: a case report

**DOI:** 10.1007/s15010-020-01457-w

**Published:** 2020-06-18

**Authors:** Judith E. Spiro, Snezana Sisovic, Ben Ockert, Wolfgang Böcker, Georg Siebenbürger

**Affiliations:** 1 Department of Radiology, University Hospital, LMU Munich, Munich, Germany; 2 Department of Internal Medicine IV-Endocrinology, Munich University Hospitals, Ludwig-Maximilians-University, Munich, Germany; 3 Department of General, Trauma and Reconstructive Surgery, Munich University Hospitals, Ludwig-Maximilians-University, Nussbaumstr. 20, 80336 Munich, Germany

**Keywords:** COVID-19, Pneumothorax, Pneumonia, Multidetector computed tomography, Viral infections

## Abstract

**Purpose:**

Especially in elderly and multimorbid patients, Coronavirus Disease 2019 (COVID-19) may result in severe pneumonia and secondary complications. Recent studies showed pneumothorax in rare cases, but tension pneumothorax has only been reported once.

**Case presentation:**

A 47-year-old male was admitted to the emergency department with fever, dry cough and sore throat for the last 14 days as well as acute stenocardia and shortage of breath. Sputum testing (polymerase chain reaction, PCR) confirmed SARS-CoV-2 infection. Initial computed tomography (CT) showed bipulmonary groundglass opacities and consolidations with peripheral distribution. Hospitalization with supportive therapy (azithromycin) as well as non-invasive oxygenation led to a stabilization of the patient. After 5 days, sputum testing was negative and IgA/IgG antibody titres were positive for SARS-CoV-2. The patient was discharged after 7 days.

On the 11th day, the patient realized pronounced dyspnoea after coughing and presented to the emergency department again. CT showed a right-sided tension pneumothorax, which was relieved by a chest drain (Buelau) via mini open thoracotomy. Negative pressure therapy resulted in regression of the pneumothorax and the patient was discharged after 9 days of treatment.

**Conclusion:**

Treating physicians should be aware that COVID-19 patients might develop severe secondary pulmonary complications such as acute tension pneumothorax.

**Level of evidence:**

V

**Electronic supplementary material:**

The online version of this article (10.1007/s15010-020-01457-w) contains supplementary material, which is available to authorized users.

## Background

Since its first description in Wuhan, Hubei Province, China, in December 2019, severe acute respiratory syndrome coronavirus 2 (SARS-CoV-2) has led to a pandemic, which was officially declared a global health emergency by the world health organization (WHO) on January 30, 2020 [1, 2]. Coronavirus disease 2019 (COVID-19) may lead to severe viral pneumonia. Characteristic computed tomography (CT) findings in affected patients include bilateral, multilobar ground-glass opacities (GGO) and consolidations with peripheral and posterior distribution [3–6]. Aside from common symptoms like dry cough, fever, myalgia and/or fatigue, severe secondary complications are described in recently published studies: acute respiratory distress syndrome (ARDS), acute kidney or cardiac injury, secondary infection and liver dysfunction [7]. Reports of pneumothorax as a complication of COVID-19 are rare, and therefore, we describe a case of secondary tension pneumothorax.

## Case report

A 47-year-old male was admitted to our emergency department with dry cough, shortness of breath and stenocardia. Body temperature on initial admission was 37.9 ℃. Due to a traumatic motorcycle accident, the patient had undergone splenectomy years ago. Because of human immunodeficiency virus (HIV) infection, the patient was under treatment with Dovato^®^ 50/300 mg (GSK^®^, Dolutegravir/Lamivudine). Nadir CD4 count was 573 cells/µL (23% of lymphocytes) and lowest CD4/CD8 ratio 0.47 2 days after hospitalization compared to 1210 cells (29% of lymphocytes) and a CD4/CD8 ratio of 0.74 at time of admission. One month before, at a regular check-up, CD 4 count was 1408 cells/µL (35% of lymphocytes) and CD4/CD8 ratio 0.9. The patient had no previously known pulmonary or thoracic diseases and no history of smoking. There was no recent history of travel to a designated COVID-19 risk area, but contact to an infected individual in the patients’ social environment. Symptoms including dry cough, fever, shortness of breath and stenocardia had lasted for 14 days before primary admission.

Blood gas analysis (arterial) at admission showed a pH of 7.49, PCO_2_ of 32.6 mmHg, PO_2_ of 110 mmHg, and SaO_2_ at 98.9% with 5L/min oxygen applied by nasal cannula, which was hence reduced to 3L/min oxygen, resulting in a peripheral O_2_ saturation of 95%.

Polymerase chain reaction (PCR) tests of nasal and pharyngeal swabs and sputum were positive for SARS-CoV-2-RNA N-gene 1, but negative for RSV, Influenza-A and Influenza-B. HIV-1-RNA testing (PCR) showed a level of < 40 copies/mL.

Blood samples showed increased levels of CRP (10.2 mg/d), lactate dehydrogenase (LDH) (406 U/L), leucocytes (12.4 G/L), D-dimers (1.7 µg/mL), and interleukine-6 (122 pg/mL). Procalcitonin (PCT) levels were ≤ 0.1 ng/mL. Neutrophil count was reduced to 35.0% and lymphocyte count was normal (46%).

The electrocardiogram was normal. Troponin T (hs) was negative (≤ 0.013).

CT scan at admission showed bipulmonary GGO and consolidations with a multilobar, peripheral and dorsal distribution. Pulmonary embolism, suspected due to elevated levels of d-dimers, was ruled out. Detailed information about the radiological appearance and follow-up of the patient can be found in the supplement.

Because of progressive coughing and shortness of breath, the patient received 3 mg of morphine and was admitted to the intermediate care ward. Further therapy consisted of azithromycin to prevent secondary bacterial superinfection. Antibody titres (Anti-SARS-CoV-2-IgG and Anti-SARS-CoV-2-IgA) were positive and SARS-CoV-2-RNA N-gene 1 (PCR) was negative 5 days after primary admission. The patient was discharged with lowering CRP levels as well as normalized leucocytes and interleukine-6 levels after 7 days.

Four days later, 11 days after primary admission, the patient admitted again to the emergency department with pronounced dyspnoea after coughing.

Blood gas analysis (arterial) showed a pH of 7.47, PCO_2_ of 33 mmHg, PO_2_ of 64 mmHg, and SaO_2_ of 94% with 3L/min oxygen applied by nasal cannula. Clinical elevation showed a reduced right sided breathing sound. The electrocardiogram showed tachycardia (103 bpm).

**Fig. 1:**
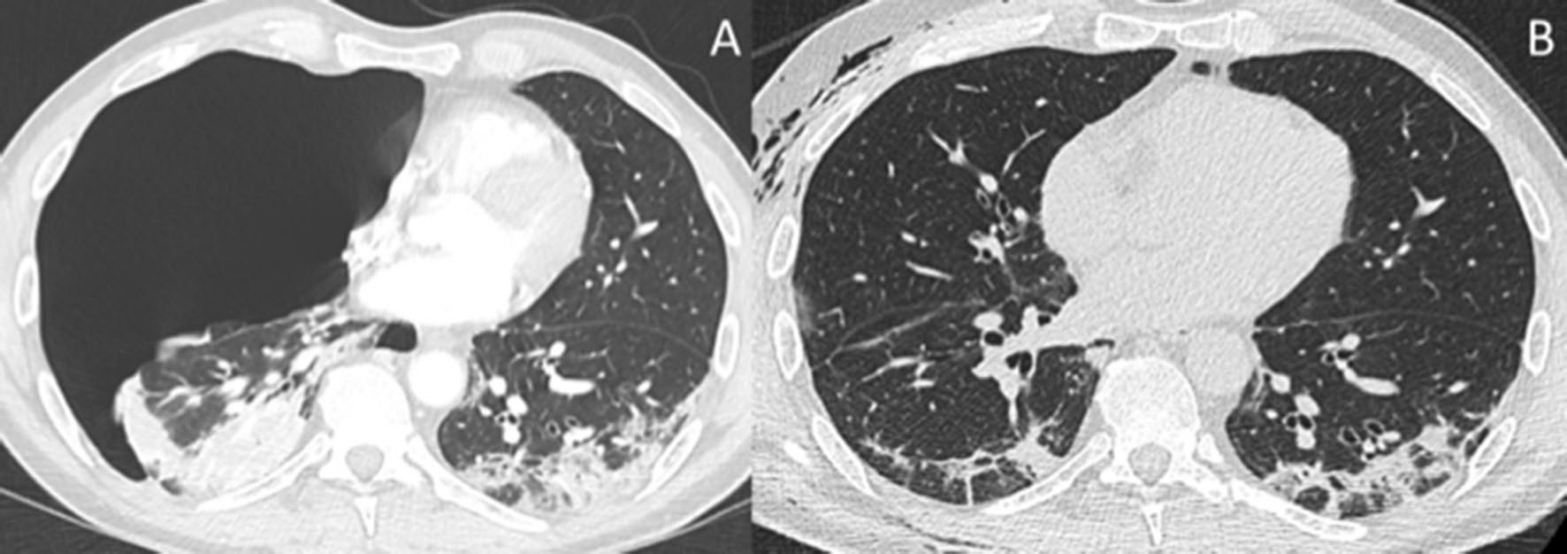
Axial CT images obtained with intravenous contrast at second admission show a right-sided tension pneumothorax with mediastinal shift to the left and right heart compression (**a**). Unenhanced axial CT images obtained 8 days after second admission show remission of tension pneumothorax, new soft tissue emphysema of the right chest wall and mild pneumomediastinum (**b**). Bilateral GGO and consolidations with peripheral distribution on both images are signs of COVID-19 pneumonia. CT, computed tomography; GGO, ground glass opacities

Thoracic CT revealed a right-sided tension pneumothorax (Fig. 1a). To relieve the tension pneumothorax, a small caliber chest tube (20 Charrière = French, Buelau) was inserted through a mini open thoracotomy in the 5th right intercostal space. Due to secondary displacement, the drain had to be repositioned 4 h later. Continuous negative pressure (− 20 cm/H_2_O) led to almost complete regression of the pneumothorax. Five days later, clogging of the chest drain by a blood clot caused brief worsening of the patient’s status with relapse of the pneumothorax and increasing soft tissue emphysema. When the clot was removed, the pneumothorax resolved again. Follow-up CT 8 days after second admission confirmed good re-expansion of the right lung, residual soft tissue emphysema of the right chest wall and mild pneumomediastinum (Fig. 1b).

The chest tube was removed after 8 days and the patient was discharged after 9 days (20 days after primary admission) in stable condition.

**Fig. 2:**
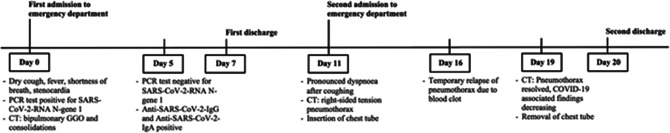
Timeline of the patient history from first admission to second discharge. COVID-19, Coronavirus Disease 2019. CT, computed tomography. GGO, ground glass opacities. PCR, polymerase chain reaction. RNA, ribonucleic acid. SARS-CoV-2, severe acute respiratory syndrome coronavirus 2

A timeline of the events is presented in Fig. 2.

## Discussion

Its relatively high sensitivity and availability have made CT of the chest an important screening tool for COVID-19, especially when PCR testing capacities are limited. Characteristic CT findings include pulmonary GGO and consolidations, often with a bilateral, dorsal and subpleural distribution. Additionally, thoracic CT plays an important role in the follow-up of COVID-19 patients, because it allows evaluation of the course of disease as well as detection of acute complications, such as pulmonary embolism and pneumothorax [8–10].

To date, spontaneous pneumothorax has been seldom reported in patients with COVID-19. In one case from Wuhan, formation of subpleural bullae was observed on chest CT as a possible cause for development of mediastinal emphysema and pneumothorax [9]. Our patient, however, did not show pulmonary cysts or bullae on CT scans, neither before onset, nor after treatment of tension pneumothorax. Tension pneumothorax after infection with SARS-CoV-2 has been described only once before in a recently published case report by Flower et al. [11] Larger studies showed a pneumothorax prevalence of 1–2% in adults with COVID-19 [4, 7, 12, 13].

Spontaneous pneumothorax is a commonly known complication in patients with ARDS, where the most frequent causes are pressure and volume-related alveolar rupture [14]. Histological examination of lung biopsy samples in a patient who died from COVID-19 pneumonia showed desquamation of pneumocytes and hyaline membrane formation, indicating ARDS [7]. Before onset of severe dyspnea, our patient suffered from coughing, which increases alveolar pressure. Considering the absence of pre-existing pulmonary conditions as well as a negative smoking history, in this case we suspect the pneumothorax to be caused by the structural lung injury following a COVID-19 pneumonia.

## Conclusion

We present a rare but severe complication of infection with SARS-CoV-2. The case of secondary tension pneumothorax underlines the importance of prompt and thorough clinical evaluation of COVID-19 patients with worsening respiratory status.

## Electronic supplementary material

Below is the link to the electronic supplementary material.

Supplementary Figure 1: Axial CT images obtained with intravenous contrast at first admission show bilateral GGO and consolidations with peripheral and dorsal distribution (a-c) as well as hilar lymphadenopathy (d). CT, computed tomography; GGO, ground glass opacities. (JPEG 108 kb)

Supplementary Figure 2: Axial CT images obtained with intravenous contrast at second admission show a right-sided tension pneumothorax (a-c) with mediastinal shift to the left and right heart compression (d). Compared to CT at first admission, bilateral GGO and consolidations have decreased in size in increased in density (slices a-c, obtained at the same levels as in Figure 1). CT, computed tomography; GGO, ground glass opacities. (JPEG 87 kb)

Supplementary Figure 3: Chest radiographs in posteroanterior view. Tension pneumothorax was relieved by insertion of a chest tube, which secondarily displaced to the chest wall (a). After repositioning of the drain to the pleural space, the pneumothorax almost completely resolved under continuous negative pressure; note mild soft tissue emphysema of the right chest wall (b). Five days later, clogging of the chest drain by a blood clot caused relapse of the pneumothorax and progression of soft tissue emphysema (c). When the clot was removed, the pneumothorax resolved again (d) (JPEG 55 kb)

Supplementary Figure 4: Unenhanced axial CT images obtained 8 days after second admission show remission of tension pneumothorax (a-c), soft tissue emphysema of the right chest wall (a-d) and mild pneumomediastinum (d). Compared to CT at second admission, intrapulmonary GGO and consolidations have further decreased in size and increased in density and linear opacities are visible in the dorsal subpleural areas of both lungs (slices a-c, obtained at the same levels as in Figure 1 and 2). CT, computed tomography; GGO, ground glass opacities. (PNG 399 kb)

## Data Availability

All available information is contained within the manuscript.
