# Supplementary figures and images for: Secondary tension pneumothorax in a COVID-19 pneumonia patient: a case report
Source: Infection. 2020 Jun 18;48(6):941–4. doi: 10.1007/s15010-020-01457-w (PMC7301769; doi:10.1007/s15010-020-01457-w)

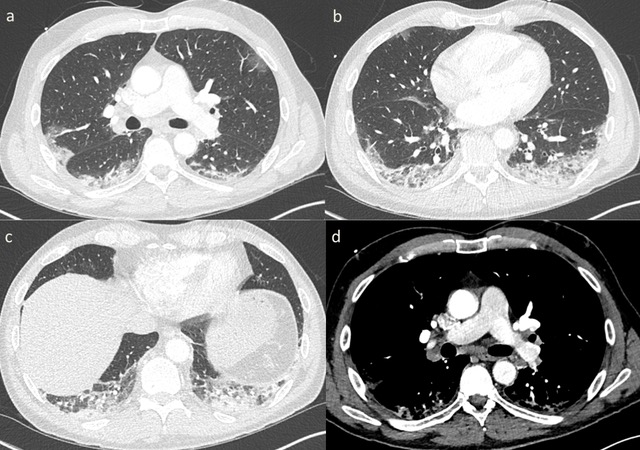

Supplement: Supplementary file 1 — Supplementary Figure 1: Axial CT images obtained with intravenous contrast at first admission show bilateral GGO and consolidations with peripheral and dorsal distribution (a-c) as well as hilar lymphadenopathy (d). CT, computed tomography; GGO, ground glass opacities. (JPEG 108 kb) [file 15010_2020_1457_MOESM1_ESM.jpg]

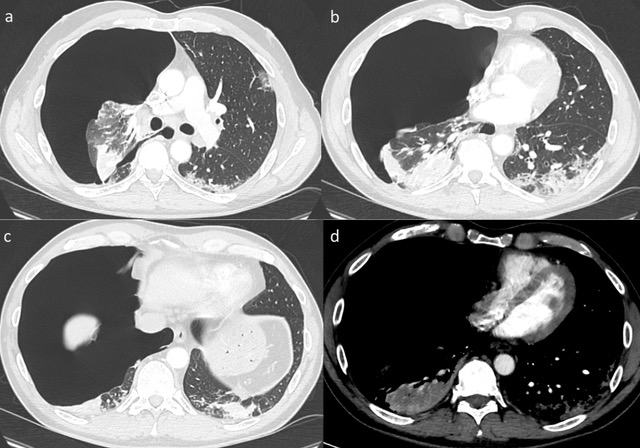

Supplement: Supplementary file 2 — Supplementary Figure 2: Axial CT images obtained with intravenous contrast at second admission show a right-sided tension pneumothorax (a-c) with mediastinal shift to the left and right heart compression (d). Compared to CT at first admission, bilateral GGO and consolidations have decreased in size in increased in density (slices a-c, obtained at the same levels as in Figure 1). CT, computed tomography; GGO, ground glass opacities. (JPEG 87 kb) [file 15010_2020_1457_MOESM2_ESM.jpg]

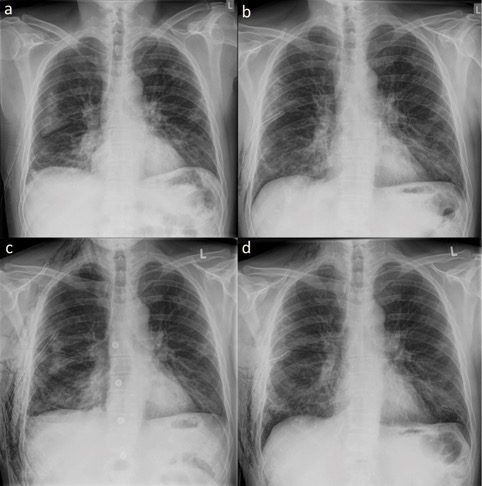

Supplement: Supplementary file 3 — Supplementary Figure 3: Chest radiographs in posteroanterior view. Tension pneumothorax was relieved by insertion of a chest tube, which secondarily displaced to the chest wall (a). After repositioning of the drain to the pleural space, the pneumothorax almost completely resolved under continuous negative pressure; note mild soft tissue emphysema of the right chest wall (b). Five days later, clogging of the chest drain by a blood clot caused relapse of the pneumothorax and progression of soft tissue emphysema (c). When the clot was removed, the pneumothorax resolved again (d) (JPEG 55 kb) [file 15010_2020_1457_MOESM3_ESM.jpg]

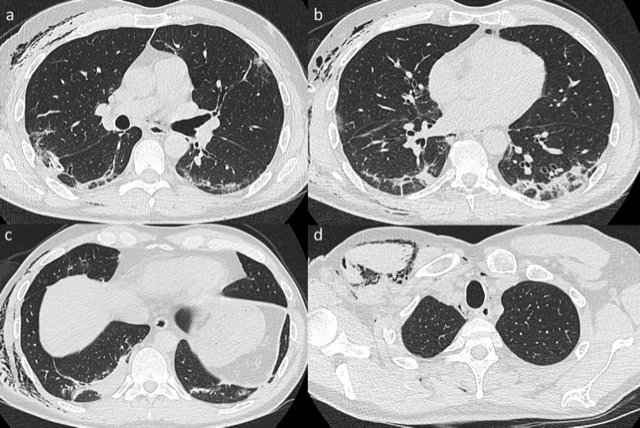

Supplement: Supplementary file 4 — Supplementary Figure 4: Unenhanced axial CT images obtained 8 days after second admission show remission of tension pneumothorax (a-c), soft tissue emphysema of the right chest wall (a-d) and mild pneumomediastinum (d). Compared to CT at second admission, intrapulmonary GGO and consolidations have further decreased in size and increased in density and linear opacities are visible in the dorsal subpleural areas of both lungs (slices a-c, obtained at the same levels as in Figure 1 and 2). CT, computed tomography; GGO, ground glass opacities. (PNG 399 kb) [file 15010_2020_1457_MOESM4_ESM.png]
